# Forskolin Suppresses Delayed-Rectifier K^+^ Currents and Enhances Spike Frequency-Dependent Adaptation of Sympathetic Neurons

**DOI:** 10.1371/journal.pone.0126365

**Published:** 2015-05-11

**Authors:** Luis I. Angel-Chavez, Eduardo I. Acosta-Gómez, Mario Morales-Avalos, Elena Castro, Humberto Cruzblanca

**Affiliations:** 1 Departamento de Ciencias de la Salud, Instituto de Ciencias Biomédicas, Universidad Autónoma de Ciudad Juárez, Ciudad Juárez, Chih. 32310, México; 2 Centro Universitario de Investigaciones Biomédicas, Universidad de Colima, Colima, Col. 28045, México; Dalhousie University, CANADA

## Abstract

In signal transduction research natural or synthetic molecules are commonly used to target a great variety of signaling proteins. For instance, forskolin, a diterpene activator of adenylate cyclase, has been widely used in cellular preparations to increase the intracellular cAMP level. However, it has been shown that forskolin directly inhibits some cloned K^+^ channels, which in excitable cells set up the resting membrane potential, the shape of action potential and regulate repetitive firing. Despite the growing evidence indicating that K^+^ channels are blocked by forskolin, there are no studies yet assessing the impact of this mechanism of action on neuron excitability and firing patterns. In sympathetic neurons, we find that forskolin and its derivative 1,9-Dideoxyforskolin, reversibly suppress the delayed rectifier K^+^ current (I_KV_). Besides, forskolin reduced the spike afterhyperpolarization and enhanced the spike frequency-dependent adaptation. Given that I_KV_ is mostly generated by Kv2.1 channels, HEK-293 cells were transfected with cDNA encoding for the Kv2.1 α subunit, to characterize the mechanism of forskolin action. Both drugs reversible suppressed the Kv2.1-mediated K^+^ currents. Forskolin inhibited Kv2.1 currents and I_KV_ with an IC_50_ of ~32 μM and ~24 µM, respectively. Besides, the drug induced an apparent current inactivation and slowed-down current deactivation. We suggest that forskolin reduces the excitability of sympathetic neurons by enhancing the spike frequency-dependent adaptation, partially through a direct block of their native Kv2.1 channels.

## Introduction

In signal transduction research, a plethora of natural or synthetic molecules have been currently used as pharmacological tools to search for the nature of intracellular pathways that underlies specific cellular processes [1]. For instance forskolin (FSK), an activator of adenylate cyclase, is frequently applied to cellular preparations to study cAMP-dependent transduction pathways [2,3,4,5]. Nevertheless, early evidence shows that FSK may have cAMP-independent effects on ligand- or voltage-gated ion currents [6,7,8]. Indeed, it is well known that FSK directly blocks a variety of cloned K^+^ channels including the Kv1.1 and Kv1.4 α subunits [9], a Na^+^-activated K^+^ channel [10] and the TRESK background K^+^ channel [11]. In excitable cells, voltage-gated K^+^ channels generate the resting membrane potential, shape the action potential and regulate the firing pattern [12,13]. So far there are no studies in excitable cells exploring the effect on repetitive firing of the FSK-mediated K^+^ channel block.

Superior cervical ganglion (SCG) neurons are a suitable cellular model because in these sympathetic cells are well known those K^+^ currents contributing to the resting potential, shaping the action potential and regulating firing frequency. For instance, the M-type K^+^ current (I_KM_) contributes to the resting potential and its inhibition, either by G_q/11_-coupled receptors or drugs, enhances the probability of repetitive firing [14,15]. Moreover, other voltage-gated K^+^ currents contribute to regulate the firing pattern, including: 1) the fast activating and inactivating A-type K^+^ current (I_A_); 2) a second type of I_A_ with slower inactivation (I_As_) and; 3) the delayed rectifier K^+^ current I_KV_ [16]. The relative level of current density among these broad kinetic types of K^+^ currents, settle the firing pattern of SCG cells [16,17,18]. Thus, rat SCG neurons can be classified as phasic, adapting and tonic cells [16,18,19]. Besides, there are subtle differences at a molecular level because in SCG neurons expressing I_A_ and I_KV_, the latter is generated by homomeric Kv2.1 and Kv2.2 channels, whereas Kv2.1 channels mostly contribute to I_KV_ in those nerve cells expressing I_A_, I_As_ and I_KV_ [17]. Here, we find that FSK mostly suppresses both I_KV_ and Kv2.1-mediated K^+^ currents and enhances the spike frequency-dependent adaptation of SCG neurons.

## Material and Methods

### 2.1 Cell culture and transfection procedures

Experiments were performed on cultured SCG neurons from 4-weeks old male Wistar rats. Animal procedures were approved by the Universidad de Colima ethics and biosecurity committee. Briefly, rats were decapitated under anesthesia and the ganglia were removed and placed in a Ca^2+^-free Hank´s solution (37°C) containing papain (20 U/ml). Thereafter, papain was replaced by a mixture of collagenase I (1.6 mg/ml) and dispase II (5 mg/ml). Mechanically dissociated cells were suspended twice in DMEM, centrifuged and plated onto poly-L-lysine-coated glass chips. Cells were incubated at 37°C (5% CO_2_) with DMEM supplemented with 10% of heat-inactivated fetal bovine serum (FBS). For other experiments, HEK-293 cells (Life Technologies, Carlsbad, CA) were grown in DMEM supplemented with 5% FBS in the incubator and passaged every 3 days. Cells were transiently co-transfected with rat Kv2.1 and GFP cDNA, with lipofectin according to the manufacture’s recommendations. The next day, cells were plated onto the glass chips and GFP-positive cells were recorded 8–24 hrs later on.

### 2.2 Electrophysiology and data analysis

Action potentials were recorded with the whole cell current-clamp technique, using the HEKA-10 amplifier running with the patch-master software (HEKA Instruments, Southboro, MA). Action potentials were evoked by depolarizing current pulses (100 to 250 pA, 1500 ms) and the spikes were filtered at 10 KHz. Whole-cell records of I_KV_ or Kv2.1 currents were obtained with an EPC-7 amplifier (HEKA Instruments, Southboro, MA). Seals were obtained with patch pipettes having 1–2 MΩ resistance and current recordings began 2 min after seal breakthrough, once stable series resistance (~3 MΩ) was reached. In HEK-293 cells (mean membrane capacitance and input resistance were 11.4 ± 1.2 pF and 200 ± 31 MΩ, respectively) series resistance was compensated (50–70%) to improve voltage-clamp control. Command pulses and K^+^ current records were generated and acquired (sampling rate, 5 kHz) using a 12 bit interface (Indec Systems Inc. Sunnyvale, CA). The holding potential was -50 mV and K^+^ currents were activated by 100 ms pulses from -40 mV to +40 mV. The tails of both I_KV_ and Kv2.1 currents were recorded during 100 ms, upon return to -50 mV from 0 mV. For I_KV_ and Kv2.1 current quantification four traces obtained from each experimental condition were computer averaged, then the tail current amplitude was measured as the difference between the average of a 2 ms current segment (10 data points), taken at the beginning of the tail current, and the average during the last 5 ms (25 data points) of the current record. The software BASIC-FASTLAB (Indec Systems Inc. Sunnyvale, CA) and Sigma Plot (SPSS Inc. Chicago IL) were used to analyze the K^+^ currents. In some experiments, the N-type Ca^2+^ current (I_Ca_), Na^+^ current (I_Na_), I_KM_ or I_A_, were recorded separately. I_Ca_ and I_Na_ were recorded with a Cs-based pipette solution and elicited by a 10 ms and 4 ms command pulses, respectively, from -80 mV to +10 mV. I_KM_ and I_A_ were recorded and analyzed as described [20,21]. The percent of current suppression was calculated as [1-(current _test_/current _control_)] x 100%. Statistics are given as the mean ± s.e.m. and sample means were compared for significance with the Student’s t test (p ≤ 0.05).

### 2.3 Solutions and chemicals

Cells were transferred to a recording chamber (400 μl) and bathed (2.8 ml/min) with the appropriate external solution. Solution changes were accomplished in ~ 10 s and experiments were done at ~25°C. The external solution for K^+^ current recording was (in mM): 160 NaCl, 2.5 KCl, 5 CaCl_2_, 1.0 MgCl_2_, 10 HEPES and 8 glucose (pH = 7.4, adjusted with NaOH). To block the neuronal I_Na_ and I_Ca_, tetrodotoxin (0.5 μM) and Cd^2+^ (200 μM) were added, respectively, to the external solution. The internal solution used for SCG neuron recording was (in mM): 175 KCl, 5 MgCl_2_, 5 HEPES, 0.2 BAPTA, 3 K_2_ATP, 0.1 Na-GTP, 0.08 leupeptin (pH = 7.4, adjusted with KOH). The pipette solution for HEK-293 cells was (in mM): 140 KCl, 2 MgCl_2_, 10 HEPES, 0.2 BAPTA, 3 K_2_ATP, 0.1 Na-GTP, 0.08 leupeptin (pH = 7.3, adjusted with KOH). The osmolarities for the SCG and HEK-293 internal solutions were 321 mOsm and 290 mOsm, respectively (WESCOR Inc. Logan, Utah). Stock solutions (20 mM) of FSK and 1,9-Dideoxyforskolin (1,9-dFSK) were prepared with DMSO. Therefore the range of concentration of DMSO in the external solution was between 0.00005% and 0.005%.

Chemicals were purchased as follows: Collagenase I, poly-L-lysine, HEPES, Na-GTP, 1,9-dFSK (Sigma, St. Louis, MO); BAPTA (Molecular Probes, Eugene, OR); papain, dispase II, leupeptin and K_2_-ATP (Roche Diagnostics GmbH, Mannheim, Germany); DMEM, FBS and Lipofectin (Life Technologies, Carlsbad, CA); TTX, FSK and 8-Br-cAMP (Calbiochem, La Jolla, CA).

## Results

### 3.1 Forskolin predominantly suppresses I_KV_ in rat sympathetic neurons

**Fig 1:**
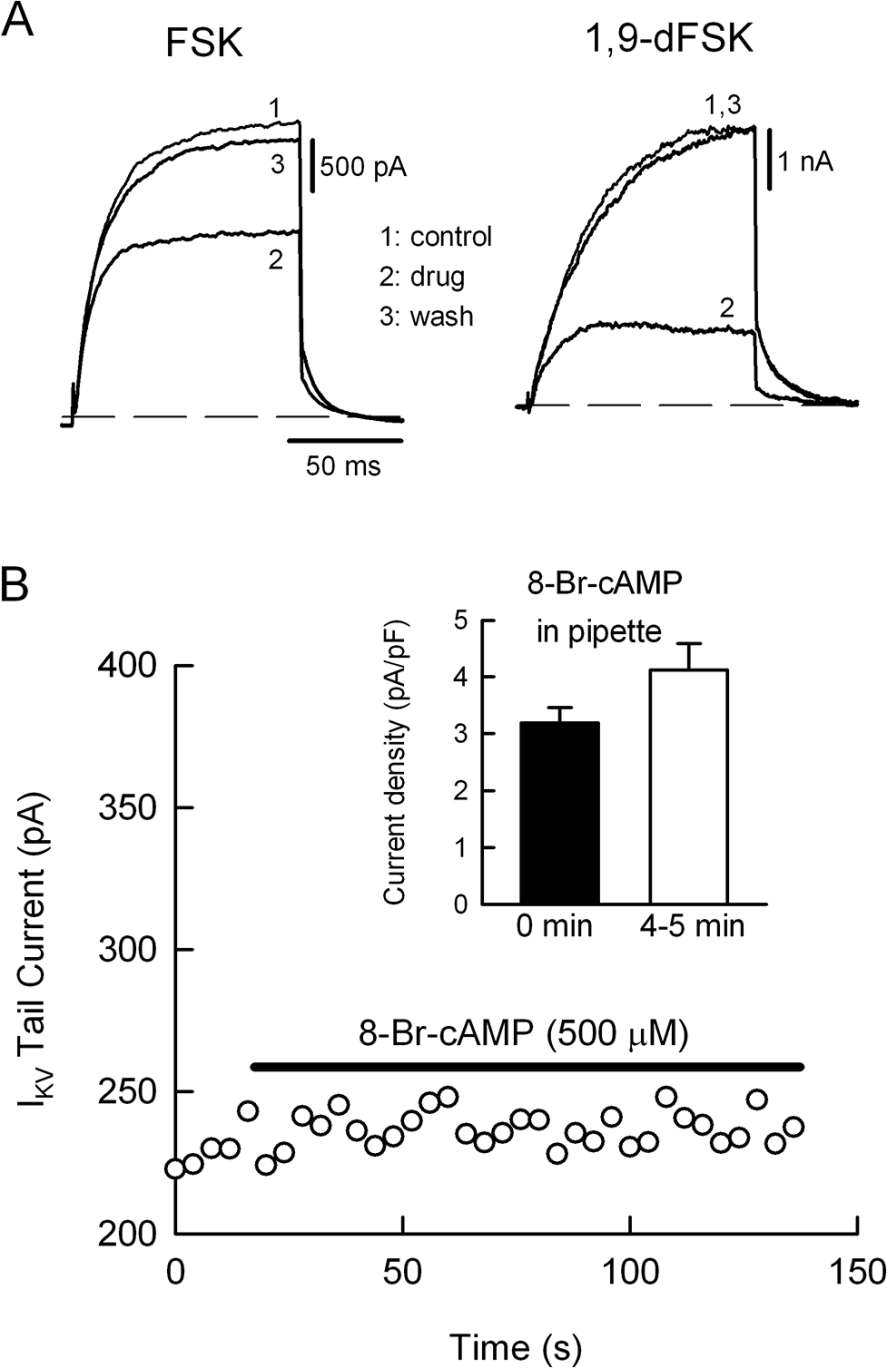
Forskolin reduces the delayed rectifier K^+^ current I_KV_ independently of cAMP. (A) Representative traces of I_**KV**_ recorded before (1), during the maximum effect (2) of 20 μM of FSK (left panel) or 1,9-dFSK (right panel), and after drug washout (3). The dashed line indicates zero current level. (B) The I_**KV**_ tail current amplitude was plotted every 4 s and the horizontal bars indicate the period of time to the external exposure of 8-Br-cAMP (500 μM). Note that the membrane permeable analogue of cAMP does not change the amplitude of the tail current. The inset summarizes the results obtained when 8-Br-cAMP was supplied through the patch pipette. The bars represents the mean value (± s.e.m., n = 4) of the tail current density upon seal breakthrough (filled bar, time zero) and 4–5 min after dialysis with the K^**+**^-standard internal solution containing 8-Br-cAMP (open bar). The tail current density slightly increased from 3.2 ± 0.2 pA/pF to 4.1 ± 0.4 pA/pF upon cell dialysis, nevertheless, the enhancement was not statistically significant.

**Fig 2:**
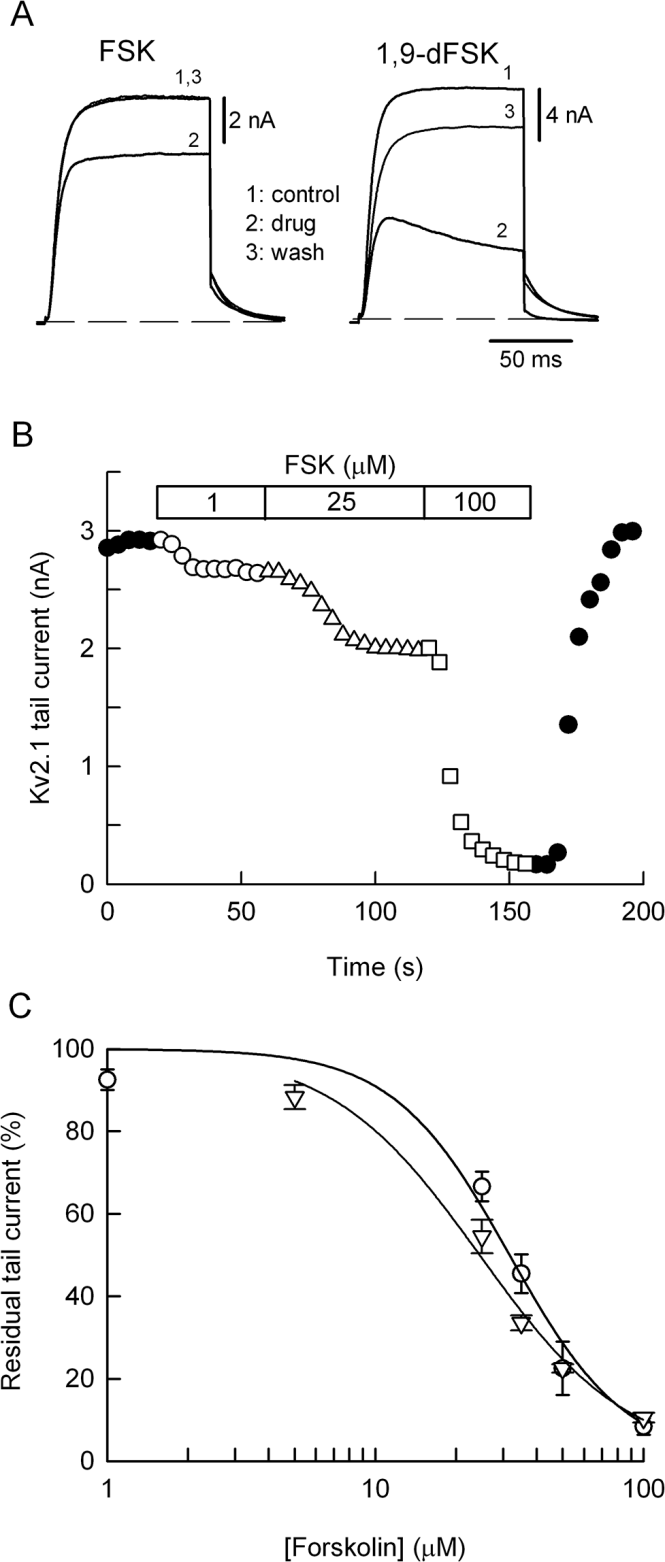
Forskolin reduces Kv2.1 K^+^ currents and I_KV_ in a concentration-dependent manner. (A) Kv2.1 currents recorded before (1), in the presence (2) of 20 μM FSK (left panel) or 1,9-dFSK (right panel), and after drug washout (3). (B) Kv2.1 tail current was measured every 4 s, in the absence (filled circles) or with 1 μM (open circles), 25 μM (open triangles) and 100 μM (open squares) of FSK. Note the full recovery of the K^**+**^ current after drug washout. (C) Percent of the remaining tail current generated by the deactivation of Kv2.1 channels (circles) or I_**KV**_ (triangles), at various drug concentrations. Data points were fitted to the equation Y = 100-[100/(1+(IC_**50**_/FSK)^**n**^)], where n is the Hill coefficient. The fits yield the values of n = 2 and IC_**50**_ of 31.6 μM for the Kv2.1 current (3–9 cells for each concentration), whereas for I_**KV**_ the values are n = 1.6 and IC_**50**_ of 24.4 μM (8–14 cells).

In SCG neurons muscarinic or angiotensin II receptors enhance I_KV_ through a pertussis toxin-insensitive G-protein [20,22]. To assess if the cAMP pathway underlies the enhancement of I_KV_, cells were transiently exposed to 20 μM FSK. In contrast with the receptor action, FSK reversibly suppressed I_KV_ and this effect was completely mimicked by 1,9-dFSK, the derivative molecule without action on adenylate cyclase (Fig 1A). To confirm that suppression of I_KV_ was independent of cAMP synthesis, SCG neurons were challenged with 500 μM of 8-Bromo-cAMP (8-Br-cAMP). The membrane permeable analogue of cAMP had no effect on I_KV_, either when the drug was externally applied or through the patch pipette (Fig 1B). The inhibitory effect of FSK on I_KV_ was concentration-dependent at the range of 5–100 μM, thus yielding an IC_50_ of 24.4 μM (triangles in Fig 2C). We further ask if other SCG endogenous voltage-gated ion currents are regulated by FSK. Neither I_Na_ nor I_Ca_ was considerably inhibited by 100 μM FSK, yielding a mean suppression of 6.1 ± 3.3% and 6.6 ± 0.9%, respectively, whereas I_KM_ was slightly reduced by 12.8 ± 1.2%. In contrast, I_KV_ and I_A_ were suppressed by 89.7 ± 1.4% and 53.4 ± 0.8%, respectively. Data were taken from 4–6 neurons for each type of ionic current.

### 3.2 Forskolin and 1,9-dFSK reduce Kv2.1 K^+^ currents in HEK-293 cells

We decided to focus our study on I_KV_ because yet it was strongly suppressed by FSK and this effect was independent of cAMP synthesis. To quantify the inhibitory potency of FSK and to assess the mode of drug action, we switched our experiments to HEK-293 cells transfected with cDNA encoding for the Kv2.1 K^+^ channel α subunit, because: a) in SCG neurons homomeric Kv2.1 channels mostly contribute to I_KV_ [17] and; b) this approach avoids possible current contamination by others native voltage-gated K^+^ channels. Similarly to our findings in SCG neurons, both FSK and 1,9-dFSK reversibly reduced the Kv2.1-mediated K^+^ currents (Fig 2A). To measure the inhibitory potency of FSK, cells were transiently challenged with increasing drug concentrations, randomly selected at the range of 1–100 μM. FSK reduced the tail current amplitude with an IC_50_ of 31.6 μM (circles in Fig 2C), while 100 μM FSK produced a mean suppression of 91 ± 2% (n = 9). It is noteworthy that even with the highest concentration used there was a relatively fast and full recovery of the K^+^ current upon drug removal (Fig 2B).

**Fig 3:**
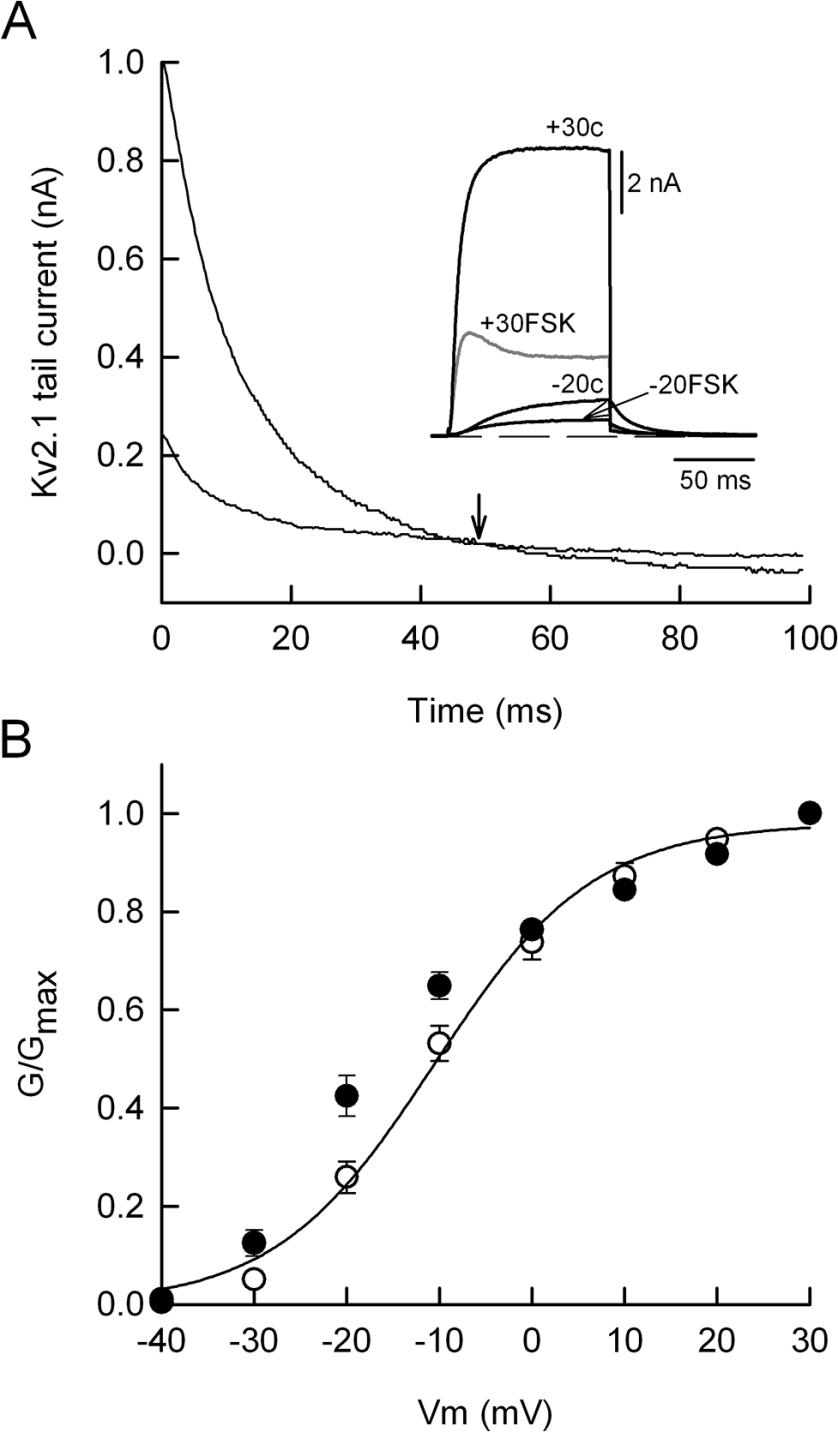
Effects of forskolin on Kv2.1 channel deactivation and its steady-state activation. (A) The inset shows representative Kv2.1 currents elicited by membrane depolarization to -20 mV or +30 mV (V_**h**_ = -50 mV), before (c) and in the presence of 35 μM forskolin (FSK). Note that at +30 mV FSK induced a conspicuous “inactivation” of the Kv2.1 current. The tail currents recorded at +30 mV are expanded to reveal the tail current crossover provoked by FSK (downward arrow). (B) Steady-state activation curve of Kv2.1 current before (open circles) and in the presence of FSK (filled circles). Data points represent the mean of four cells (± s.e.m.) for each experimental condition. The continuous line indicates the fit to the Boltzmann equation only to the control data. Mean values for V_**1/2**_ and the slope factor are described in the main text.

FSK produced changes on K^+^ current kinetics as shown in the inset of Fig 3A. In the absence of the drug the 100 ms depolarizing stimulus generates non-inactivating Kv2.1 currents at -20 mV (-20c) and +30 mV (+30c). However, with FSK there was a noticeable current “inactivation” at +30 mV. An “inactivation” of the Kv2.1 current was produced by 1,9-dFSK as well (Fig 2A), suggesting that both drugs plug the Kv2.1 channel pore after its opening. This type of open channel block is known to slow down the rate of current deactivation, thereby leading to the crossover of the tail currents [23,24]. Indeed, the current crossover was revealed when the control current and the suppressed Kv2.1 current were superimposed (arrow in Fig 3A). A more detailed analysis of the effect of FSK on current deactivation was performed by using the K^+^ current records derived from the concentration-response relationship (circles in Fig 2C), namely those obtained with 25 μM (n = 6) and 50 μM (n = 6), and fitting the tail currents to a single exponential. Thus, with 25 μM of FSK the time constant of deactivation (τ) was consistently increased from 14.7 ± 1.9 ms to 19.4 ± 2.3 ms and returned to 16.5 ± 2 ms after drug removal. As expected, 50 μM of FSK produced a grater and statistically significant (p ≤ 0.05) increase of τ from 15.5 ± 2.2 ms to 32.8 ± 4 ms, and returning to 18 ± 1.3 ms after drug washout. In contrast, FSK did not shift the steady-state activation curve of Kv2.1 channels (Fig 3B). That is, the half-activation potential (V_1/2_) and slope factor values for the control condition were -10.3 ± 1.5 mV and 8.5 ± 0.5 mV (n = 4), respectively, whereas with FSK (35 μM) the corresponding values were -16.4 ± 1.6 mV and 8.4 ± 0.5 mV (n = 4). For both parameters the difference between the control and test condition was not statistically significant.

### 3.3 Forskolin enhances the spike frequency-dependent adaptation of SCG neurons

**Fig 4:**
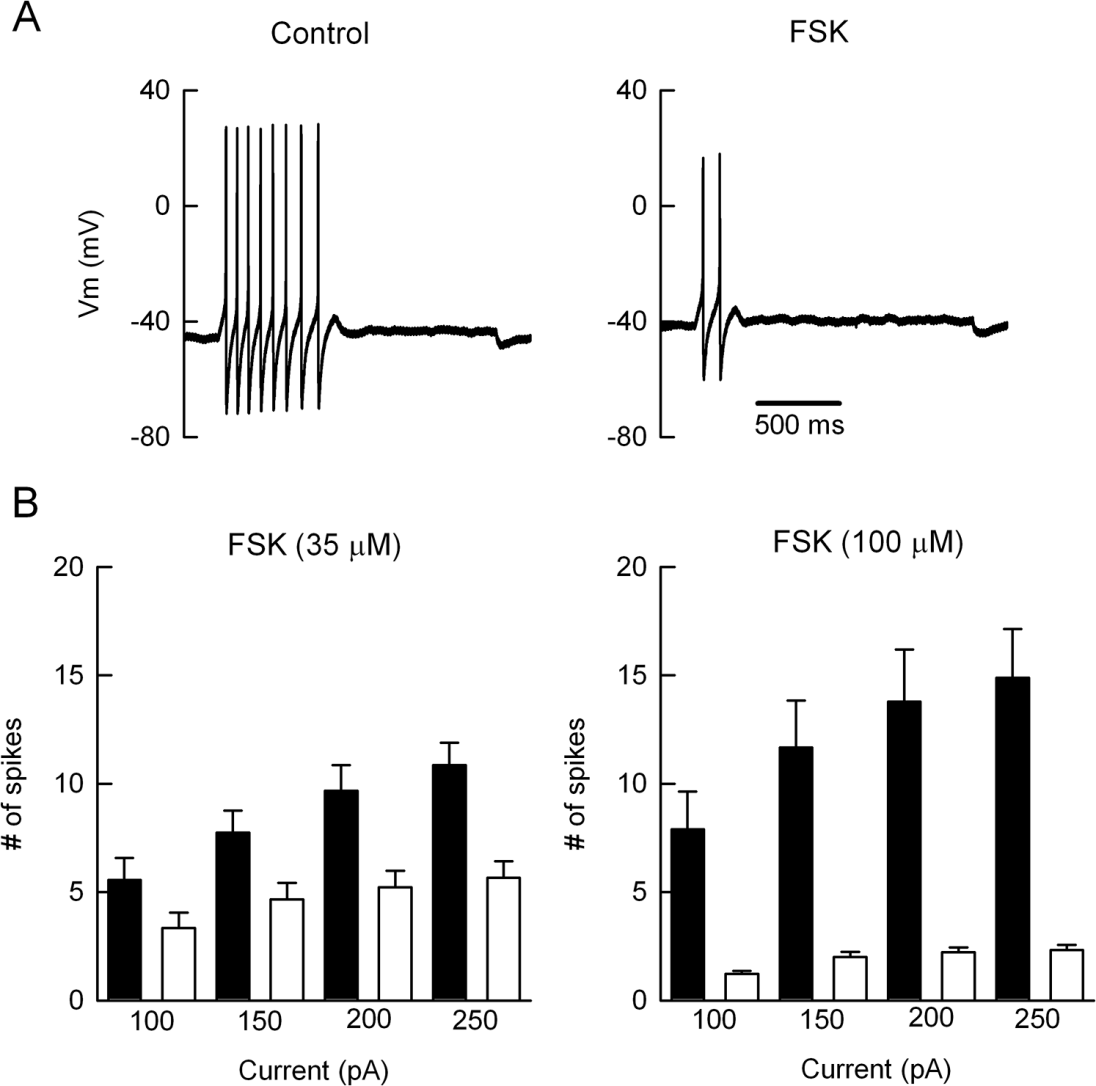
Forskolin reduces the excitability of SCG neurons. (A) Whole-cell current-clamp recordings of action potentials generated by depolarizing current injection (150 pA), before (control) and in the presence of 100 μM of FSK. In this adapting-type neuron FSK reduced the number of spikes from 8 to 2. (B) Mean number of spikes elicited by increasing stimuli amplitudes, before (filled bars) and during bath application of FSK (open bars). The reduction in the number of spikes by 35 μM of FSK (n = 20 neurons) was statistically significant for the 150 pA (p ≤ 0.5), 200 pA (p ≤ 0.01) and 250 pA (p ≤ 0.001) current stimuli. Moreover, with 100 μM of FSK (n = 11 cells) the differences were statistically significant for the 100 pA (p ≤ 0.05), 150 pA (p ≤ 0.05), 200 pA (p ≤ 0.001) and 250 pA (p ≤ 0.001) depolarizing stimuli.

In neurons voltage-gated K^+^ currents have a major role in setting the firing pattern by shaping the action potential and determining the interspike interval [12,13]. In SCG neurons I_KV_ contributes to the late phase of the action potential repolarization and to the early phase of spike afterhyperpolarization (AHP) [25]. Therefore, we sought whether suppression of I_KV_ could affect the spike firing properties of SCG cells. On the basis to their response to depolarizing current stimuli, adult rat SCG neurons can be classified as phasic (fire one action potential), adapting (fire from two to eight spikes) and tonic cells [16,18,19]. We used adapting-type neurons because they are more abundant (54%) than tonic ones (10%) [19], and in these neurons I_KV_ is mostly mediated by Kv2.1 subunits [17]. Fig 4A shows the response of an adapting neuron to depolarizing current. As expected, the cell showed spike frequency-dependent adaptation because it fired 8 action potentials at the beginning of the 1500 ms current stimulus (not shown). However, in the presence of 100 μM FSK the neuron fired only 2 and more spaced action potentials, revealing an enhancement of spike adaptation. The effect of FSK on excitability was concentration-dependent: for instance, the mean number of spikes in response to a 250 pA stimulus was reduced from 11.8 ± 1.1 to 5.9 ± 0.8 with 35 μM of FSK (n = 20 cells), and from 15.1 ± 2.4 to only 2.3 ± 0.2 in neurons challenged with 100 μM FSK (n = 11 cells). Thus, the reduction in the mean number of spikes was statistically significant for 35 μM (p ≤ 0.001) and 100 μM FSK (p≤ 0.001). Indeed, the decrease in excitability by 100 μM FSK was statistically significant for the other current amplitudes tested (Fig 4B).

**Fig 5:**
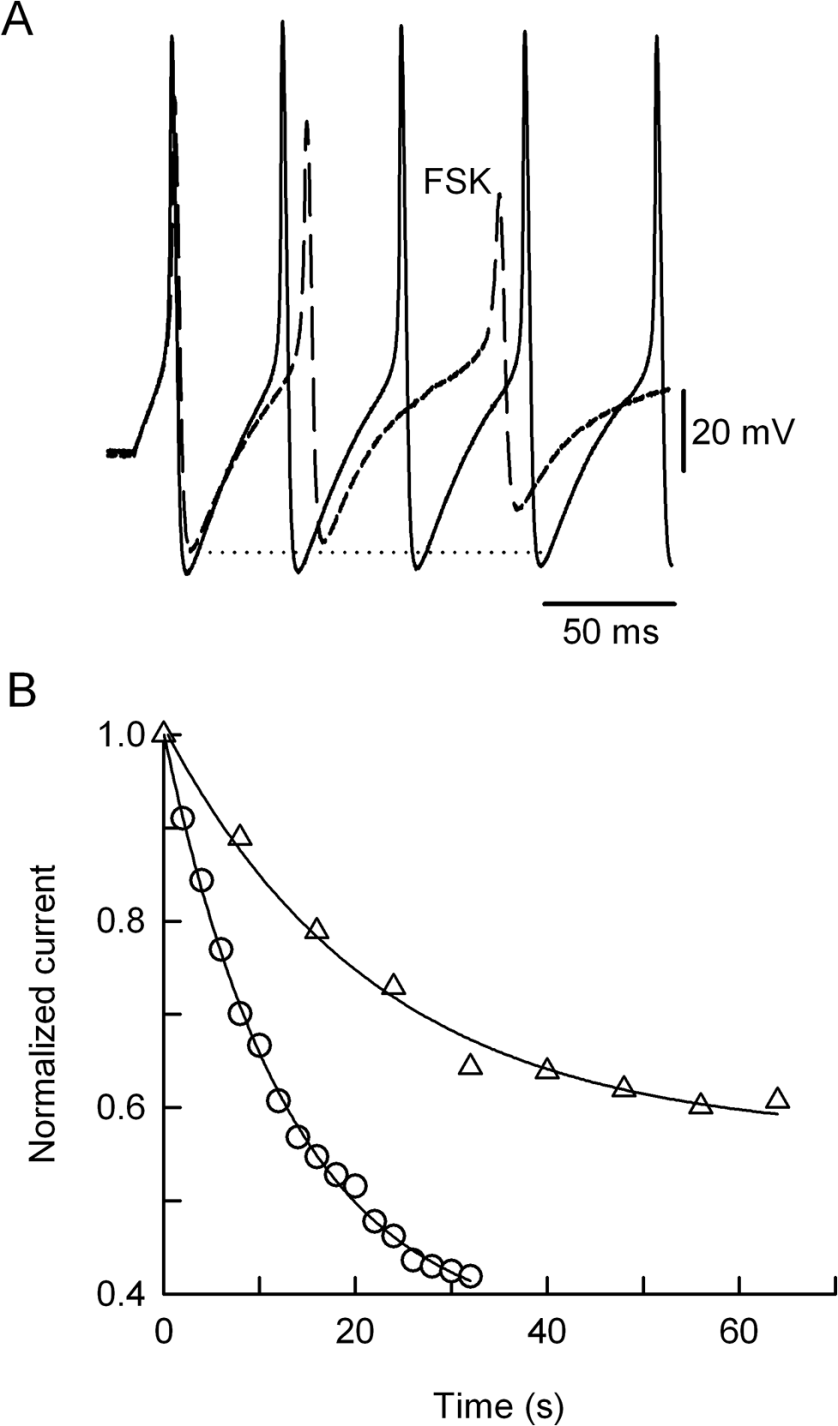
Frequency-dependent effects of FSK on AHP and Kv2.1 current. (A) Evoked action potentials in the absence (black traces) or presence of 100 μM of FSK (dashed traces). Note that FSK produced a gradual decrease of the AHP, relative to that elicited during the first action potential (dotted line). (B) Data from a representative experiment in which the Kv2.1 channels were activated every 2 s (circles) or 8 s (triangles), and the resulting K^**+**^ currents recorded during FSK exposure, were normalized to the respective control value (time = 0 s). Note that FSK (35 μM) suppressed the Kv2.1 current, faster (τ = 13 s) at 0.5 Hz than to the lower frequency of stimulation (τ = 22 s). Therefore, in six cells the time constant for Kv2.1 current suppression was increased from 9.6 ± 1.5 s to 21.8 ± 3 s, as the frequency of depolarization decreased.

It was found that FSK (100 μM) had no effect on the latency to the first spike (control = 115.1 ± 0.8 ms; FSK = 116.3 ± 0.8 ms), rather the drug increased the spike interval between the first and second action potentials from 44.9 ± 2.3 ms to 61.8 ± 6 ms (see Fig 5A). Therefore, estimating the initial firing frequency from these spike interval values it was found that FSK (100 μM) produced a statistically significant (p ≤ 0.05) decrease in the firing frequency from 22 Hz to 16 Hz. As expected, FSK also affected the spike waveform because there were a gradual broadening of the action potentials and a progressive decrease in the AHP (Fig 5A). In summary, in eleven neurons the control peak amplitude of the third AHP was 25.9 ± 0.6 mV, whereas it was significantly (p≤ 0.05) reduced to 11.7 ± 2.6 mV by FSK in four neurons, whilst the rest of the cells (n = 7) failed to generate more than two spikes. We ask if the progressive decrease of AHP would reveal some sort of used-dependent inhibition of the native K^+^ channels generating I_KV_, because the FSK-induced Kv2.1 current inactivation and its slowed deactivation (Fig 3A) are signatures of an open-channel block mechanism [13,23,24]. Indeed, FSK decreased Kv2.1 currents faster and stronger as the frequency of the depolarizing command pulse increased from 0.125 Hz to 0.5 Hz (Fig 5B). Thus, in six cells the time constant of current inhibition was significantly (p≤ 0.05) increased from 9.6 ± 1.5 s to 21.8 ± 3 s, as the frequency of depolarization decreased.

## Discussion

Here we found that in SCG neurons FSK reduces the number of spikes in response to current injection, enhances the spike frequency-dependent adaptation, reduces spike AHP and suppresses I_KV_. The robust decrease in excitability was not primarily due to suppression of voltage-gated inward currents because FSK had no significant effect neither Na^+^ nor N-type Ca^2+^ currents (Fig 1), and even 100 µM FSK neither prevented the generation of the first action potential nor affected the latency to the first spike. Rather, FSK might act on those K^+^ currents regulating repetitive firing in SCG neurons, such as I_A_, I_KM_, I_KV_ and/or I_AHP_. We suggest that neither I_A_ nor I_KM_ are responsible for the enhancement of spike adaptation because: a) the genetic suppression of the Kv4.2 α subunit, which in SCG neurons generates I_A_, decrease the percentage of phasic neurons [16], while FSK promoted a phasic-like firing phenotype (Fig 4); b) I_KM_ suppression promotes tonic firing [14,15]. A mention apart deserves the K^+^ currents contributing to the AHP.

In phasic-type SCG neurons Ca^2+^-activated SK channels generate a slow I_AHP_ after Ca^2+^ entering the cell via N-type Ca^2+^ channels [26]. Conversely, adapting-type neurons develop a shorter AHP (~ 40 ms) indicating that I_KV_ has a more significant role generating the AHP [18,25]. However, FSK might reduce I_AHP_ either by blocking the Ca^2+^ entry or inhibiting the SK-type K^+^ channels. We discard these mechanisms because FSK had no effect on I_Ca_ (Fig 1) and inhibition of SK channels with apamin increases repetitive firing of SCG cells [26]. Therefore, we propose that the FSK-induced decrease of AHP is due to inhibition of the native Kv2.1 channels because: a) the IC_50_ for Kv2.1 current and I_KV_ suppression were quite similar (Fig 2C), confirming the genetic evidence that in adapting neurons Kv2.1 channels mostly generate I_KV_ [17]; b) the phasic-like phenotype induced by FSK agrees with the parallel tonic phenotype seen upon over-expression of Kv2.1 subunits in SCG neurons [17]. The gradual decrease of AHP would be consistent with the use-dependent block of Kv2.1 channels, as seen in HEK-293 cells. However, because the different time scales between the frequency-dependent Kv2.1 suppression and the rate of AHP inhibition, further experiments are needed to confirm this proposal.

It was suggested that FSK reduces a Kv2.1-mediated delayed rectifier K^+^ current (I_K_) in cerebellar granule neurons through the cAMP/protein kinase A pathway, because: i) inhibition of I_K_ was partially attenuated either by a Kv2.1 siRNA or the protein kinase A inhibitor H-89 and; ii) the effect of FSK was mimicked by dibutyryl cAMP [27]. Thus, there are major differences between the mechanism used by FSK to suppress the cerebellar I_K_ and that involved in inhibition of I_KV_ and Kv2.1 currents. For instance, 50 μM FSK reduced I_K_ by 30–35% [27], whereas the same concentration strongly inhibited Kv2.1 currents and I_KV_ by the same amount (~ 77%) (Fig 2C). A third difference concerns with the recovery of K^+^ currents upon FSK washout, that is, recovery of I_K_ was slow and incomplete (see Fig 4 in [27]); in comparison recovery of I_KV_ and Kv2.1 currents was fast and complete (Figs 1 and 2). Therefore, in our experimental conditions we do not think that the cAMP/protein kinase A pathway is the principal player in the strong action of FSK on I_KV_ or Kv2.1 currents. First, 1,9-dFSK reversibly reduced both I_KV_ and Kv2.1 K^+^ currents. Second, 8-Br-cAMP, applied externally or through the patch pipette had no effect on I_KV_ (Fig 1B). Third, FSK did not change the V_1/2_ for Kv2.1 currents (Fig 3B), ruling out a mechanism of channel phosphorylation because this type of modulation shifts the V_1/2_ of Kv2.1 channels to depolarized potentials [28,29]. Instead, we favor an open-channel mechanism of blockage, because: a) the speed and strength of current suppression was use-dependent (Fig 5B); b) FSK induces a tail current crossover indicating that after their opening, Kv2.1 channels deactivate slowly relative to the drug-free condition (Fig 3A) and c) FSK and 1,9-dFSK induce a partial current “inactivation” during the 100 ms command pulse (Fig 2A and inset in Fig 3A). In agreement with our results, it was reported that both FSK and 1,9-dFSK block Kv1.1 and Kv1.4 homomeric channels by an open-channel mechanism [9].

Kv2.1 channels are the major molecular correlate of the delayed rectifier K^+^ current in central neurons [30,31,32]. In hippocampal CA1 neurons, the genetic suppression of Kv2.1 subunits results in action potential broadening and longer interspike intervals, when Schaffer collaterals are stimulated at 1 Hz but not at lower frequencies [33]. Besides, it has been suggested that the spike AHP limits Na-channel inactivation and hence facilitating high frequency repetitive firing of hippocampal neurons [34]. Therefore, we suggest that in SCG neurons the enhancement of the frequency-dependent adaptation primarily occurs when FSK suppresses I_KV_ and the hyperpolarizing drive mechanism is compromised, thereby favoring sodium channel inactivation and longer interspike intervals. In the absence of the drug, the adapting type neurons fired action potentials at a frequency ~ 20 Hz (Fig 5A) suggesting that I_KV_ regulate high frequency firing as well.

## Conclusions

It is concluded that FSK reduces the excitability of SCG neurons and enhances the spike frequency-dependent adaptation, and this effects could be partially attributed to direct inhibition of native Kv2.1 channels. The fact that the IC_50_ for Kv2.1-current (~32 μM) and I_KV_ (~24 μM) suppression are in the range of the EC_50_ (25 μM) for the FSK-induced elevation of cAMP in rat cerebral cortical slices [35], and that FSK block Kv2.1 currents by an open-channel mechanism, suggests that this drug may affect the neuronal firing pattern independently of the cAMP signaling pathway.
